# The complete chloroplast genome of *Hippuris vulgaris* (Plantaginaceae)

**DOI:** 10.1080/23802359.2020.1860706

**Published:** 2021-01-28

**Authors:** Dongmei Liu, Lijuan Li, Pengju Liu

**Affiliations:** a The Institue of Ecology, Chinese Research Academy of Environmental Sciences, Beijing, China; b Wuhan Botanical Garden, Chinese Academy of Sciences, CAS Key Laboratory of Plant Germplasm Enhancement and Specialty Agriculture, Wuhan, Hubei, China; c CAS Key Laboratory for Plant Diversity and Biogeography of East Asia, Kunming Institute of Botany, Chinese Academy of Sciences, Kunming, Yunnan, China

**Keywords:** Chloroplast genome, *Hisppuris vulgaris*, phylogeny

## Abstract

*Hippuris vulgaris* is an aquatic perennial herb distributed worldwide. In this research, the complete chloroplast genome of *H. vulgaris* was sequenced and assembled. Its complete genome size was 152,698 bp in length. The typical quadripartite structure was shown, which contained a large single-copy region (82,940 bp), a small single-copy region (18,262 bp), and a pair of inverted repeat regions (25,748 bp). The CG content of this genome was 37.6%. A total of 114 genes have been identified in the genome, including 80 protein-coding genes, 30 tRNA genes, and 4 rRNA genes. In addition, 18 genes possessed at least one intron. The phylogenetic analysis indicated that *H. vulgaris* was nested in Plantaginaceae with 100% bootstrap value and was a sister to *Digitalis*, *Plantago*, *Hemiphragma*, *Veronica* and *Veronicastrum*.

*Hippuris vulgaris* L. is an aquatic plant of worldwide distribution mainly occurred in circumboreal regions with creeping rhizomes, single stem and heterophyll (Chen JR et al; Chen JM et al. 2013). The plant grows in streams, lakes, paddy fields, river shores and bogs from 40 m to 5000 m above sea level (Chen JR 2000; Lu et al. 2016). To date, the complete plastid genome of *Hippuris* has not been identified. Hence, we sequenced, assembled and annotated the chloroplast genome of *H*. *vulgaris*, and tried to ascertain the phylogenetic status of this genome.

Fresh leaves of *H*. *vulgaris* were collected from Zaduo, Qinghai, China (N32°47′54.59″, E95°8′55.18″). A specimen as the voucher is deposited in the KUN (Herbarium, Kunming Institute of Botany, Chinese Academy of Sciences; deng7287). *Hippuris vulgaris* was sequenced by the high-throughput sequencing method with the help of Beijing Novartis Bioinformatics Technology Co., Ltd. The paired-end reads obtained after sequencing were assembled using NOVOPlasty v. 3.7.1 (Dierckxsens et al. 2017). The assembled sequence was annotated in MPI-MP CHLOROBOX (https://chlorobox.mpimp-golm.mpg.de/index.html) via GeSeq (Tillich et al. 2017) with 2 reference genomes (*Plantago depressa* Willd. and *Plantago lagopus* L.), and then manually corrected using Geneious v.9.0.2 (Kearse et al. 2012). Eventually, the complete chloroplast genome of *H. vularis* was submitted to Genbank (GenBank accession number is MT942637).

The genome of *H*. *vulgaris* is 152,698 bp in length, which is divided into four parts, including a large single-copy (LSC) region of 82,940 bp, a small single-copy (SSC) region of 18,262 bp and two inverted repeat (IR) regions of 25,748 bp. The CG contents of LSC, SSC, IR and whole genome are 35.7%, 30.8%, 43.1% and 37.6%, respectively. There are 114 unique genes annotated, including 80 protein-coding genes, 30 tRNA genes and 4 rRNA genes. Among them, 6 protein-coding genes, 7 tRNA genes and 4 rRNA genes are duplicated in the IR regions. Of the 18 genes containing introns, three of them have two introns (*clpP*, *ycf3* and *rps12*).

The phylogeny of *H. vulgaris* was analysis based on the maximum likelihood (ML) method. A total of 11 genomes used for analysis were obtained from NCBI (National Center for Biotechnology Information Search database; https://www.ncbi.nlm.nih.gov/), of which *Buddleja alternifolia* Maxim. and *Scrophularia buergeriana* Miq. were selected as outgroups. We aligned the genome with the support of mafft v7.308 (Katoh et al. 2002). By using BioEdit v.7.0.5.3 (Hall 1999), we checked and corrected the alignment. The nucleotide substitution model was tested by ModelFinder (Kalyaanamoorthy et al. 2017). In accordance with the Akaike Information Criterion (AIC), GTR + F + I + G4 was identified as the best appropriate model. The phylogenetic analysis was performed using 1000 replicates in IQ-TREE v.1.6.12 (Trifinopoulos et al. 2016). FigTree v.1.4.4 was used to view the phylogenetic tree. The results indicated that *H. vulgaris* was nested in Plantaginaceae with very strong support (100% bootstrap value). Furthermore, this species was a sister to *Digitalis*, *Plantago*, *Hemiphragma*, *Veronica* and *Veronicastrum* (Figure 1).

**Figure 1.**
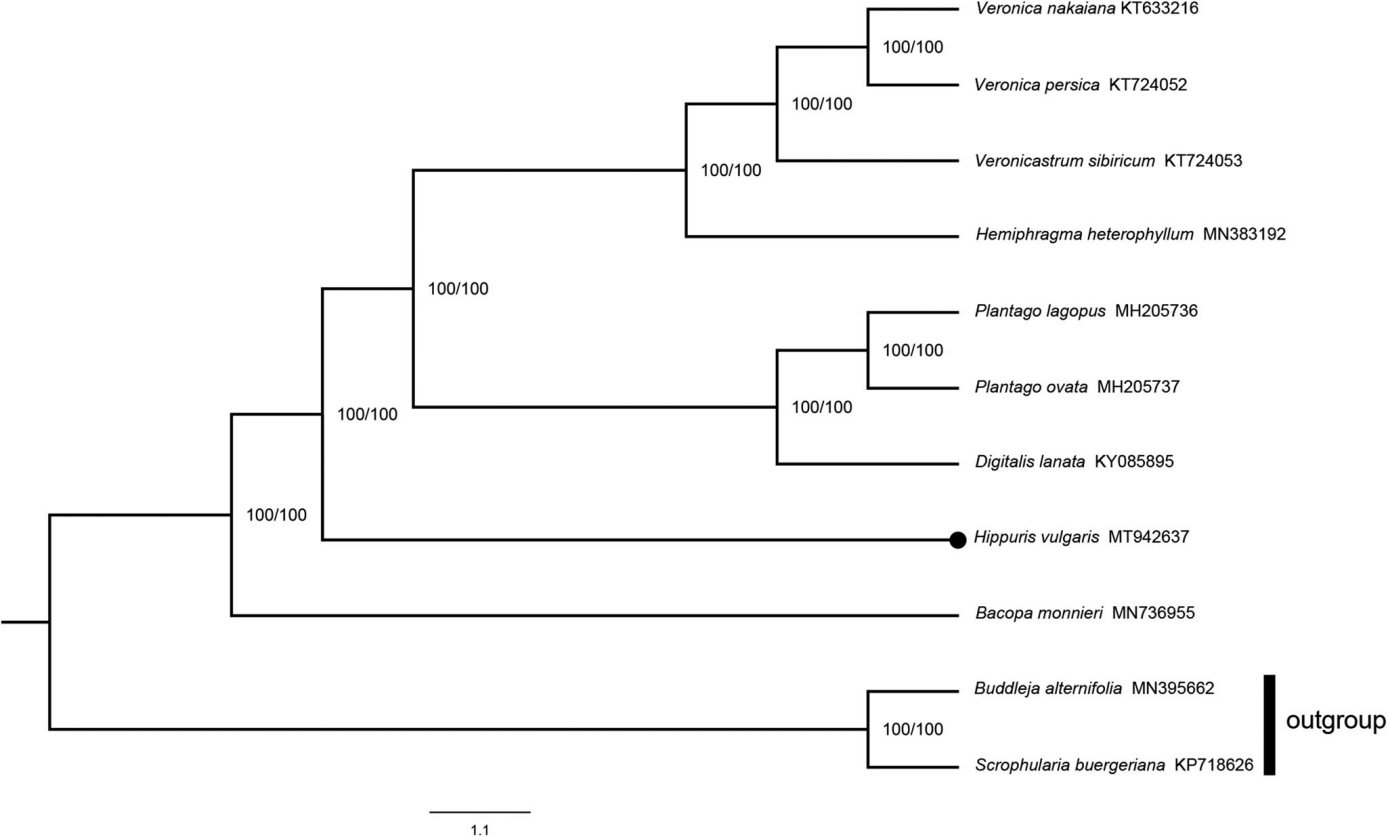
The ML phylogenetic tree based on 11 whole genomes. The numbers at the notes are SH-aLRT support (%)/ultrafast bootstrap support (%) from 1000 replicates. The black dot indicates *Hippuris vulgaris*.

## Data Availability

The data that support the findings of this study are openly available in GenBank at https://www.ncbi.nlm.nih.gov/, reference number MT942637, and in Sequence Read Archive at https://trace.ncbi.nlm.nih.gov/, reference number SRX9471852.
